# The current evidence based medical management of vesicoureteral reflux: The Sickkids protocol

**DOI:** 10.4103/0970-1591.36714

**Published:** 2007

**Authors:** Sumit Dave, Antoine E. Khoury

**Affiliations:** Division of Urology, The Hospital for Sick Children and University of Toronto, Toronto, Ontario, M5G 1X8, Canada

**Keywords:** Vesico-ureteral reflux, urinary tract infection, nephropathy

## Abstract

Vesicoureteral reflux is a common clinical entity and is one of the keystones of the establishment of pediatric urology as a urological subspeciality. There has been continued evolution in the management of vesicoureteral reflux as new insights are gained on its role in renal damage. The optimal treatment algorithm remains controversial. This review aims to highlight the current literature on VUR and its association with urinary tract infections and renal damage. The protocol of management of a child with VUR followed at The Hospital for Sick Children, Toronto is described.

## INTRODUCTION

The management of vesicoureteral reflux (VUR) has undergone a paradigm shift from open surgical correction at diagnosis in the 70's to conservative medical management over the next 2 decades and a resurgent interest in early correction using endoscopic therapy in the recent years. The primary reason for treating VUR as a disease entity has been its association with urinary tract infection (UTI), specifically pyelonephritis (PN), leading to renal scarring and subsequent hypertension and/or end stage renal failure.[1–2] This perception that the triad of UTI-VUR-nephropathy is an intimate link has driven physicians to actively diagnose and treat VUR over the last 3 decades. Interestingly, this association has never been systematically proven and on the contrary there have been several recent studies, which have demonstrated that renal scarring can occur without the presence of VUR.[3–5] Primary VUR occurs in less than 1% of the general population and as many as 50% of children who present with a UTI will have VUR.[6,7] Therefore, the detection of VUR is abnormal but there is increasing realization that it is more a marker of a generalized urinary tract abnormality rather than a defect solely of the ureterovesical junction mechanism. It represents a spectrum of pathology, which includes the associated renal dysplasia or hypoplasia, bladder dysfunction and a possible predisposition to UTI.

The primary objective of management of VUR is prevention of renal injury. The present article reviews the medical management of primary VUR as practiced at the Hospital for Sick Children, Toronto and discusses the relevant evidence for the current practice guideline. It must be emphasized that several upcoming prospective studies and the AUA VUR guidelines update (2008) may further alter the management.

## DIAGNOSIS OF VUR

VUR is diagnosed in 4 possible situations: following a UTI, in newborns with antenatally detected hydronephrosis (HN), in asymptomatic siblings who are identified after reflux is diagnosed in the proband and in situations wherein VUR is likely as in children with multicystic dysplastic kidneys (MCDK), renal agenesis and pelvic kidneys. The first 2 categories of VUR represent different nosological entities with differences in epidemiological characteristics, upper tract changes, type of bladder dysfunction and VUR resolution rates [Table 1].

**Table 1:** Differences in antenatally detected and post urinary tract infection vesicoureteral reflux

Characteristics	Antenatally detected vesicoureteral reflux	Post urinary tract infection vesicoureteral reflux
Gender	Males	Females
Severity	Usually bilateral high grade	Less severe
Dysplasia	Often severe, global	Less frequent, focal
Resolution	High resolution rate	Less likely
Bladder dysfunction	High voiding pressures, dyssynergia	Overactivity, frequent association with LUTS

### Diagnosis following first UTI

The American Academy of Pediatrics Committee on Quality Improvement recommends a voiding cystourethrogram (VCUG) for all children aged between 2 months to 2 years old following the first febrile UTI.[8] For older children, age, gender, race, type (febrile or non febrile) and frequency of UTI must be considered before proceeding to a VCUG. Toilet trained female children are primarily evaluated by a full voiding diary and the dysfunctional voiding system score (DVSS) rather than a VCUG.[9] However in the presence of a well documented episode of PN or recurrent febrile UTI we would proceed to a VCUG. All males with a febrile UTI should undergo a VCUG.

The VCUG is an invasive test associated with significant distress to the parent and discomfort to the child. There is also a real iatrogenic risk of UTI and urethral trauma in infants. Radiation exposure is another emerging concern. The timing of the VCUG following an episode of UTI was traditionally 4-6 weeks to allow the inflammatory changes in the bladder time to settle as they may potentially alter the dynamics and competency of the UVJ and the distal ureter. It has been however shown that this is not true and a VCUG can be reliably performed once the infection has resolved.[10]

There is also emerging debate regarding the role of the DMSA scan Vs. the VCUG as the first investigative modality following a UTI. The primary reason for investigating children with UTI is to detect risk factors for further renal damage. In 303 children under 2 years of age evaluated with VCUG and DMSA scans after an episode of UTI, Hansson *et al*. found that 51% had an abnormal DMSA scan and 46% with a positive DMSA scan had no evidence of VUR on VCUG.[11] There was a significant association between ≥ grade III VUR and DMSA positive renal lesions. Dilating VUR with a normal DMSA scan was found in only 7 children in this study, of whom, only 1 showed a renal scarring on subsequent follow up. None of the 7 children had recurrent UTI on follow up. The authors suggested that DMSA could replace VCUG as the primary evaluation for children following a UTI. VCUG 's could be selectively performed in children with abnormalities on DMSA scans and thus reducing the number of VCUG's by about half based on the results of this study. An early DMSA scan would identify almost all children with grade III VUR and all children with grade IV- V VUR. Mingin *et al*. retrospectively reviewed records of children who underwent DMSA scans following a febrile UTI or antenatal HN.[12] 88% of the children with an abnormal DMSA scan had grade III-V VUR. Of the 51 children with an abnormal DMSA and grade III-V VUR 60% had subsequent breakthrough UTI. In comparison only 6% of children with similar VUR grade and a normal DMSA scan developed breakthrough infection. In contrast in 63 children with normal DMSA scans and VUR the incidence of subsequent UTI was 8%. DMSA scans are generally accepted to be more sensitive than US scans in identifying renal scarring. However as shown in a study by Merguerian *et al*., US scans correlated with DMSA scans in all children with diffuse renal scarring.[13] In this study of 368 patients, in only 13 (3.5%) the DMSA scan altered the management. The authors suggested selective use of DMSA to reduce costs and radiation exposure.

At Sickids, our protocol includes an initial VCUG performed 3-4 weeks after resolution of the infection. The dose of antibiotic prophylaxis is doubled a day before the test and continued at therapeutic levels for another day following the test.

### Postnatal diagnosis of antenatally detected HN

VUR is suspected antenatally in the presence of ureteric dilatation and/or HN or diagnosis of conditions like duplication anomalies, pelvic kidneys, multicystic dysplastic kidney and renal agenesis wherein there is an increased incidence of contra lateral VUR. Van Eerde *et al*., performed a meta-analysis to review the value of antenatal HN in predicting postnatal VUR.[14] HN was defined as renal pelvic diameter > 4mm with or without caliectasis. Of the 1178 cases, the mean prevalence of primary VUR was 14.9%. When stratified by antero-posterior renal pelvic diameter (APD), VUR was diagnosed in 14% of infants with APD ≤ 10 mm and in 12% of infants with APD ≥ 10 mm. It is known that a negative prenatal screening or a normal postnatal US in infants with antenatal HN does not rule out VUR. Lee *et al*. showed that unlike UPJ obstruction, the risk of VUR did not change with increasing degree of antenatal HN.[15] In a study conducted on 108 children with antenatal HN at The Hospital for Sick Children, VUR was detected in 15% and there was no correlation between the degree of pelviectasis on postnatal US and the presence or severity of VUR.[16] Children with antenatal HN and VUR have a more benign course with a higher resolution rate when compared to children diagnosed with VUR following a UTI. Upadhyay *et al*., followed 25 children with antenatally detected HN and VUR.[17] Reflux was ≥ grade III in 70% of children. VUR resolved in 52% and was downgraded in 24%. Breakthrough urinary tract infection occurred in 4 patients with grades IV and V reflux and dysfunctional voiding developed in 5. Followup renal scans showed 19% and 17% decreased differential function in 2 units without new scars.

We recommend a VCUG in the following situations for children with antenatally detected HN:

1. All children with SFU grade 3-4 HN
2. All children with bilateral HN
3. All children with ureteric dilatation
4. Children with pelvic/ectopic kidneys and all children with a high anorectal malformation.

The role of evaluating for contra lateral VUR in multicystic dysplastic kidneys (MCDK) is controversial. The prevalence of VUR in children with MCDK ranges from 13-28% and between 5-24% in unilateral renal agenesis.[18–20] In the largest series looking at contra lateral VUR associated with MCDK, Miller *et al*., found a 25% prevalence with about half of these being low grade (I-II) and the majority showed spontaneous resolution with only 1 of 75 patients requiring surgical intervention.[18] Renal agenesis is associated with a higher incidence of VUR and shows a lower spontaneous resolution rate as compared to MCDK with contra lateral VUR.[20]

## SIBLING VUR

Primary VUR is familial and is inherited as a Mendelian dominant with partial expression, the gene frequency being 1 in 600.[21] Several studies on sibling VUR have identified factors, which can help predict the risk of sibling VUR:

1. Hollowell *et al*., showed that 44% of siblings < 2 years of age have VUR as compared to 9% of siblings > 6 years.[22]
2. If the sex of the sibling or proband is considered separately there is no statistical association. On the other hand, female siblings of the female index patient have a higher likelihood of VUR than their male counterparts.
3. Monozygotic twins have an obviously higher risk than dizygotic twins.

Characteristics of asymptomatic sibling VUR[23,24]

- Approximately one third of asymptomatic siblings of refluxers will have VUR
- 2/3rds are low grade (I, II)
- 50% unilateral
- Lower incidence of scarring/dysplasia than the index case
- Higher resolution rate

We recommend an US for initial screening for siblings. In the presence of any US evidence of scarring/dysplasia a VCUG is performed in children under 5 years of age as these form the subset of siblings most at risk of renal damage. For asymptomatic children above 5 years of age, a through evaluation is done for their voiding habits and a VCUG or RNC is performed only if there is an episode of UTI. Symptomatic siblings at any age are evaluated with a VCUG.

## MEDICAL MANAGEMENT OF VUR

It is important at this point to mention and acknowledge the important studies, which have guided VUR treatment over the last few decades. In 1960, Hodson and Edwards demonstrated renal scarring in patients with VUR, some of whom had no previous history of VUR suggesting the direct water hammer effect of VUR as a causative factor.[1] Bailey coined the term reflux nephropathy to describe the renal scarring that resulted from UTI and VUR.[25] In 1978, Ransley and Risdon showed that VUR and infection were essential pre requisites for the development of renal scars in a normal pressure system.[2] They also noted that the shape of the papillary duct orifices determined the susceptibility to intrarenal reflux.[26] Further seminal work by Smellie and others led to the guidelines for investigating and treating VUR.[27,28]

Recent studies have however questioned the role of VUR in renal scarring and in several, scarring occurred more often in the absence of VUR. Acquired renal scarring correlates best with recurrent UTI and not with VUR and primary VUR is neither sufficient nor essential for renal damage. The exceptions to this rule are secondary reflux associated with bladder outflow obstruction or high-pressure neurogenic bladders. Gordon *et al*., performed a meta-analysis to determine the value of diagnosed VUR as a predictor of renal damage in children hospitalized with UTI.[3] They evaluated 12 studies including 537 children with 1032 kidneys and showed that primary VUR was a poor predictor of renal damage in children hospitalized with UTI. A positive VCUG increased the chance of a positive DMSA scan by about 20% whereas a negative VCUG increased the chance of negative DMSA scan by 8%. The authors concluded that the VCUG could not be used as a primary screening test to detect renal parenchymal damage in children with UTI. Also infected refluxing urine does not always lead to renal scarring and that renal damage often occurs without demonstrable VUR. In another study by Taskinen and Ronholm the authors evaluated 64 hospitalized children with PN.[29] VUR grade II or higher was detected in 19% of patients and at 2 years follow up only 3 of the 12 patients with scarring had VUR. High-grade fever during initial UTI and girls older than 1 year were shown to be factors predictive of scar formation in this study.

The role of VUR, especially lower grades, as a predisposing factor for recurrent UTI is also controversial. Nuutinen and Uhari noted a higher rate of recurrent UTI's in children with grade III-V VUR in comparison with children with grade I-II VUR.[30] It is now believed that the susceptibility for recurrent UTI is more related to a defective urothelial defence mechanism and bladder dysfunction rather than associated VUR. Conway *et al*. performed a time-to-event analysis on 611 children who presented with the first UTI to determine the association between antibiotic prophylaxis and recurrent UTI and identify risk factors for resistance.[31] The factors associated with an increased risk of recurrent UTI in this study were white race, age between 3-5 years and grade IV-V VUR. Sex and grade I-III VUR were not associated with risk of recurrence. Moreover antibiotic prophylaxis was not associated with a decreased risk of recurrent UTI in a multivariable analysis but was a risk factor for antibiotic resistance among children with recurrent UTI. The rate of recurrent UTI was 12% or 0.12 per person per year in this study.

## MEDICAL VERSUS SURGICAL TREATMENT

The management of VUR has to be guided by the principles of accepting the parental expectation after providing a correct picture of the risk of VUR, its association with UTI and renal damage and its propensity to resolve over time. Very often parents are concerned about long-term antibiotic usage coupled with repeated invasive tests. However it has been noted that parental preference in the majority is towards antibiotic prophylaxis rather than open reimplantation or endoscopic correction.[32] This study showed that parents of children with VUR would prefer 5 years of prophylaxis to open surgery and 3 years of prophylaxis to endoscopic correction. The pediatric urologist has to provide a balanced and fair pros and cons scenario for each intervention and try to modulate treatment based on parental expectation and his or her own personal experience. As a first line of treatment it is reasonable to offer antibiotic prophylaxis with observation for all grades of VUR given the fact that the condition can spontaneously resolve.

It has been conclusively shown in several RCT that surgical correction of VUR does not confer any advantage in outcome of renal function over antibiotic prophylaxis [Table 2].[33–36] The 10-year results of the IRS (European arm) showed that despite a higher incidence of febrile UTI's in the medical group, the results in terms of renal growth, function and new scars were similar in both groups.[37] The medical group patients were continued on prophylaxis till either the reflux resolved or was downgraded to grade I or at the age of 8 years. Interestingly 17% of children had unsatisfactory results (persistent VUR or obstruction) following surgery, which is considerably higher rate than the 95-99% success reported in large series of ureteric reimplantation. At 10 years 52% of high- grade (III, IV) reflux had resolved, 25% was downgraded to VUR without ureteric dilatation and 23% had persistent VUR with ureteric dilatation. In a randomized controlled trial in children with bilateral severe VUR and renal scarring Smellie *et al*. showed no difference in GFR on 4 years follow up between medical and surgical management of VUR.[38] There were no new focal scars in either group but there was a progressive thinning of the parenchyma and contraction of the previously scarred areas in 15 kidneys (7 medical group and 8 surgical group). At 10 years 2 patients from either group progressed to end stage renal disease. This study proves that most children with VUR who have progressive deterioration in renal function are born with renal dysplasia rather than having acquired post infection scarring.

**Table 2:** Results of RCT on treatment of childhood vesicoureteral reflux

Study	N (Med/surg arms)	VUR grade	Protocol	Outcomes measured	Follow up	Results
IRS(European arm)[33,34]	306 (155/151)	III-IV	RCT	VUR, UTI, renal growth	5y	No difference in scarring or UTI; PN more common in medical group in medical group
BRS[35]	161 (84/77)	II-IV	RCT	VUR, UTI, scarring	65% followed for 5 y	No difference in scarring or UTI
Scott *et al*[36]	58 (25/33)	Not known	RCT	VUR, UTI, renal growth	3y	No difference in UTI

Wheeler *et al*., in a recent meta-analysis evaluated the results of combined medical and surgical treatment with medical treatment alone.[39] The end points assessed in the 964 children included the incidence of UTI's, new or progressive renal damage, renal growth, hypertension and glomerular filtration rates. The authors noted no difference in comparing the 2 modalities except for a 60% reduction in the incidence of febrile UTI's by 5 years in those treated with a combination of ureteral reimplantation and antibiotic prophylaxis versus those who received antibiotic prophylaxis alone. The authors calculated that assuming a UTI rate of 20% for children with VUR on antibiotic prophylaxis, 9 ureteric reimplantations would be required to prevent 1 febrile UTI. In conclusion the authors state, “it is uncertain whether the identification and treatment of children with reflux confers clinically important benefit…the assumption that reflux is a modifiable risk factor is not based on strong evidence from existing randomized controlled trials…” Garin *et al*., performed a randomized prospective trial comparing prophylaxis with no prophylaxis in 218 children with or without VUR who presented with PN, comparing prophylaxis with no prophylaxis.[40] The study only included patients with grade I-III VUR. No statistically significant differences were found among the groups with respect to rate of recurrent UTI, type of recurrence, rate of subsequent pyelonephritis and development of renal parenchymal scars. The overall rate of recurrent PN in this study was 5.5% and VUR did not increase the likelihood of PN. The authors concluded that at 1- year follow-up, grade I-III VUR did not increase the incidence of UTI, PN or scarring. Moreover, this does not support a role for urinary antibiotic prophylaxis in preventing the recurrence of infection and the development of renal scars. is not supported by this study. Besides compliance with taking the prophylactic antibiotic and emergence of bacterial resistance, it is possible that other factors like frequent and complete voiding may be more important factors in the prevention of UTI. Another meta-analysis published in 2007 looked at the outcome of medical (n= 329) and surgical (n= 326) management of VUR.[41] The authors noted no statistical difference in kidney growth, renal scarring and incidence of UTI's and also commented on the poor prevalence of study design dealing with publications on VUR. All the 3 RCT's dealing with VUR treatment [Table 2] nevertheless shows that surgery provides considerable protection against PN.

The AUA Pediatric Vesicoureteral Reflux Guidelines Panel therefore recommended medical management as the initial treatment for children with VUR diagnosed following a UTI in all except children more than 1 year of age with grade V VUR and older children with bilateral grade IV VUR.[42] In children with persistent VUR, surgery was recommended except for grade I-II for which there was no consensus. In the era of availability of minimally invasive endoscopic therapy for VUR correction, there has been a considerable change in the philosophy of treating VUR at some centers. This deviation from the traditional concepts of surgical management of VUR, specifically the indications for surgery, is a reflection of parental pressure and physician philosophy. The long- term efficacy and success rates of endoscopic therapy have to be balanced with the safety and efficacy of long-term prophylaxis. Also it is debatable what form of therapy leads to a lower degree of parental anxiety and a better quality of life. Analyzing cost effectiveness of the various treatment modalities is also a consideration. Benoit *et al*. created a monetary model comparing cost effectiveness of Deflux injection at diagnosis to traditional management.[43] In the scenario wherein Deflux injection is performed at diagnosis and a ureteric reimplantation is performed if the injection failed, the success rates would have to be 86.9% (for grade III), 70.8% (grade IV), 55.8 (grade V) unilateral VUR and 97.6%, 89.8% and 89.8% respectively for bilateral reflux to achieve equal cost effectiveness. Injection at diagnosis could never achieve cost effectiveness for unilateral or bilateral grade I- II VUR. Hsieh *et al*., evaluated the cost-utility of 5 different treatment algorithms (including no treatment or follow up and endoscopic treatment at diagnosis) for grade II and III VUR and showed that a non- interventional approach constituted the highest utility and least costly treatment modality.[44]

That brings us to the question: What do we truly know about VUR?

1. Scarring associated with VUR may often represent primary renal dysplasia related to abnormal ureteric bud development and metanephric blastemal induction rather than acquired renal damage related to VUR and PN.
2. There is a significant potential for spontaneous resolution of VUR and this depends on the grade, gender, bilaterality and type of VUR.
3. Excepting massive reflux, most VUR does not predispose to UTI and sterile VUR is benign.
4. VUR plus UTI does not always result in renal scarring particularly if the infection is treated promptly.
5. Although long-term antibiotic usage is relatively safe, there have been recent concerns regarding its true benefit, the emergence of resistant bacterial strains (and possible relationship to breast cancer). On the other hand, open surgical treatment of VUR has a high success rate.

It is also known that therapeutic delay in initiating treatment following a UTI is an important factor in causing renal scarring. It has been shown clinically and experimentally that if therapy starts within the first 2-3 days of the fever there seems to be an appreciable decrease in the risk of scarring.[45,46] However, at present there exists no prospective randomized study, which proves this hypothesis that early treatment of a UTI is superior to antibiotic prophylaxis.

## MEDICAL VERSUS NO TREATMENT

There is a single abstract published in 1997 children with VUR were randomized to either a no treatment group, daily prophylaxis group or prophylaxis given 3 times a week group.[47] There was no significant difference in the risk of UTI or renal parenchymal damage between each of these groups. Craig *et al*., reviewed the Australia and New Zealand Dialysis and Transplant registry between 1971 and 1998 and noted a slight increase in reflux nephropathy.[48] Contrary to popular belief the management of VUR had not made any significant impact on the etiology of ESRD.

### 1. Antibiotic prophylaxis

The administration of prophylactic antibiotics is almost universal for all children with VUR, although there is little systematic evidence for doing so besides the initial few studies reported in the 1970's. The above discussion questions the validity of prescribing antibiotic prophylaxis for VUR. Does this require a change in our current practice (which is mainly based on expert opinion and past clinical experience)? At present we choose to continue with our past practice of prescribing antibiotic prophylaxis to all children under the age of 3 years with primary VUR pending the results and recommendations of the currently underway prospective randomized controlled trials. The AUA pediatric vesicoureteral reflux guidelines panel accepted antibiotic prophylaxis as an appropriate or reasonable initial therapy for all children up to 5 years of age who have primary reflux grade I-IV.[42] There are 2 potential barriers to the success of antibiotic prophylaxis; the first is adherence and compliance to the regimen and the second is the emergence of resistance to the prophylactic antibiotic.

Long-term antibiotic prophylaxis does not necessarily prevent UTI or scarring and can be associated with bacterial resistance and other side effects. Williams *et al*., conducted a meta-analysis of the available literature to determine the efficacy of long-term antibiotics to prevent recurrent UTI in children.[49] There was a single study in this analysis, which compared the recurrence of symptomatic UTI between the antibiotic and placebo/no treatment groups.[50] This study showed almost twice as many repeat illnesses in the antibiotic group versus the placebo group. When analyzing the risk of repeat positive urine culture, antibiotics reduced this risk compared to no treatment/placebo. No side effects were reported. Two studies, which reported the risk of repeat positive urine culture for children with VUR showed a reduced risk with antibiotics when compared to those on placebo/no treatment but there was heterogeneity between the studies. Nitrofurantoin (NFT) was found to be more effective than trimethoprim in preventing recurrent UTI but patients receiving NFT were more likely to discontinue due to side effects.

A Canadian study examined over 1600 urinary isolates in about 1000 children with urinary infection and bacterial resistance rates to antibiotics like ampicillin and co-trimoxazole or both were in the range of 30%-40%.[51] The children most at risk for having resistant isolates with an odds ratio of 24:1 were children who had been on antibiotic prophylaxis for UTI. The cost of long -term antibiotics on the health care system should also be considered. Another recent study, which has caused considerable consternation in Internet savvy parents, is the reported increased risk of breast cancer reported with increasing cumulative days of antibiotic usage.[52] This study was conducted in adult females and did not include children with VUR/UTI. There is also some emerging evidence that long-term antibiotic use may increase the severity of otitis media in children and frequency of upper respiratory tract infection in adults.[53,54]

An ideal antimicrobial for prophylaxis would be one, which is effective against the majority of uropathogens, causes minimal side effects and does not lead to development of bacterial resistance. Nitrofurantoin (NFT), trimethoprim and co-trimoxazole (Septra) continue to remain the primary prophylactic agents. Though Nitrofurantoin produces more gastrointestinal side effects than trimethoprim, there is likely to be an increased usage of the drug as bacterial resistance to trimethoprim is an emerging trend in many regions. The absorption of NFT and Septra occurs high in the gastrointestinal tract and protects the intestinal flora of the colon from long exposure periods to these antibiotics.

We use NFT or co-trimoxazole as our preferred antibiotic of choice at a dose of 2 mg/kg given at bedtime. In infants the dose is divided into 2 doses of 1mg/kg each as the voiding frequency is much higher in infants and the effectiveness of this divided regimen is therefore better. Under 2 months of age, amoxicillin or cephelexin are the preferred prophylactic antibiotics. Probiotics and its beneficial effects on altering bacterial flora and decreasing the risk of UTI are emerging as a conjunct to antibiotic prophylaxis. There is considerable evidence in the adult literature about the role of probiotics and their use in children is increasing.[55]

### 2. Management of lower urinary tract symptoms (LUTS) and bladder training

Infrequent voiding, detrusor sphincter dyssynergia and constipation increase the likelihood of bacteriuria and predispose to recurrent UTI. The association of LUTS/dysfunctional elimination syndromes with recurrent UTI and lower VUR resolution rates has been demonstrated in several studies. The European arm of the IRS estimated the prevalence of detrusor sphincter dyssynergia to be 18% in children with VUR.[56] A strong association was also found between recurrent UTI and LUTS and in those children who had spontaneous VUR resolution the prevalence of LUTS was lower. Koff *et al*. showed evidence of LUTS in 43% of children with primary VUR and 77% in a subset who had recurrent UTI.[57] Chen *et al*., in a multivariable analysis of 2759 patients demonstrated a higher rate of LUTS in girls versus boys.[58] The higher rate of DES in girls was independent of UTI and VUR status. There was no association between the presence of LUTS and VUR or UTI individually but in patients with VUR and UTI the risk of LUTS almost doubled. The Dysfunctional Voiding Scoring System (DVSS) uses a simple scoring system to numerically grade LUTS.[9] It has been further validated in a subsequent study done at our institution, wherein a significant decrease in the score indicated compliance to bladder retraining and resulted in VUR resolution.[59] The treatment of LUTS involves a combination of timed voiding, management of constipation, biofeedback with or without anticholinergic and alpha- blocker therapy for detrusor sphincter dyssynergia.[60]

### 3. Follow up guidelines

The underlying principles which guide our management and follow-up of children with VUR have been modified by the current evidence which suggest that VUR is less threatening to the child and our efforts should be to minimize invasive studies like VCUG's, limit duration of antibiotic exposure and refine the indications of surgical intervention.

#### Imaging studies

There is no clear indication in the literature regarding the frequency and role of imaging studies looking at VUR status during follow up. Thompson *et al*., devised a theoretical model and conducted a retrospective study in children with primary VUR[61] diagnosed after a UTI to evaluate different strategies of follow up and its effect on antibiotic exposure and cost. The authors recommended that children with mild VUR undergo a VCUG every 2 years whereas those with moderate to severe VUR should undergo a VCUG every 3 years. A survey of the members of the American Association of Pediatrics published in 2001, 99% of the respondents indicated that they would perform a VCUG or RNC every 12-18 months in follow up.[62]

There has been a welcome initiative in minimizing radiation exposure in children, the so- called ALARA (as low as reasonably achievable) concept.[63] It is known that a traditional VCUG exposes the child to 100 times the radiation of a radionuclide cystogram (RNC). However, with new low-dose fluoroscopic methods, it is about 10 times that of a RNC.[63] The ALARA concept has sought to define the indications for performing a VCUG/RNC, as not performing the study at all is the best protection from radiation exposure. The sensitivity of RNC for detecting VUR is equal or greater than that of VCUG (except for grade I VUR). However the grading of VUR and anatomical details cannot be determined on the RNC.

The current follow up protocol at Sickkids aims to reduce the number of VCUG/RNC performed while children are on antibiotic prophylaxis basing it on the natural resolution decay curve of VUR. The initial study or the second study if the child is referred from outside with a RNC is always a VCUG. All subsequent studies, which are done at intervals of at least 18 months, are a RNC. There may be a justification in performing the second VCUG at 12 months interval after the initial study as there is a higher resolution rate in the initial 15-20 months of observation as shown by Skoog *et al*.[64] However considering the safety of antibiotic prophylaxis, we believe a period of 18 months to 2 years is acceptable for repeating VCUG's. Upper tract status is assessed with an yearly US. DMSA scans are performed at presentation if there has been history of recurrent febrile UTI's and in follow up whenever there is a febrile UTI with US or clinical evidence of PN.

#### Examination and evaluation at follow up visit:

As per the AUA Practice Guidelines, we recommend yearly follow up where the patient's height and weight are recorded. Blood pressure measurements are recorded in all patients with evidence of renal scarring. We do not perform routine cultures in asymptomatic children though the AAP survey showed that more than half of the respondents order routine urine cultures for low and high-grade reflux in asymptomatic children on follow up.[62]

A complete evaluation of voiding habits and fluid intake is recorded and the importance of adhering to a routine of frequent and complete voiding and good bowel habits is re-stressed at each visit. In uncircumcised males the foreskin is specifically examined and steroid cream is prescribed in older children with physiological phimosis. Parents are advised about the importance of seeking medical attention early if they suspect a UTI and also the importance of ensuring that a catheter specimen of urine is obtained with a full urinalysis and culture. The yearly follow up also provides an opportunity to adjust the prophylactic dose.

Any breakthrough UTI has to be investigated thoroughly to ensure the reliability of diagnosis by correlating the presenting symptoms with the methodology of urine collection, the microscopy findings and the culture report. Smellie has elucidated the interpretation of breakthrough UTI; if the organism is sensitive to the prophylactic antibiotic then the parents may have not been compliant with the antibiotic or the dose is low, if the organism is resistant to the prophylactic antibiotic either the residual bladder volume is high or the antibiotic dosage is high.[65]

#### Resolution of VUR

Persistence of VUR is more likely in high grade VUR, in children with bilateral disease (especially in Grade IV and V) and when reflux is diagnosed in the older child. The value of the VCUG and RNC in predicting VUR resolution has been studied. It has been demonstrated that when VUR occurs at less than 60% of expected bladder capacity and the reflux volume is > 2% of bladder capacity resolution is poor.[66,67] The IRS study showed that resolution of high grade VUR continued nearly consistently through 10 years of follow up.[37] Resolution was significantly associated with grade III versus grade IV, unilateral versus bilateral and age >/ 5 years at entry versus <5 years. Neither gender nor renal scarring at entry affected resolution of VUR.

### 4. Role of circumcision

There is a role of circumcision in preventing UTI in children with high grade VUR. Sing-Grewal in a systematic review noted that assuming a 30% risk of recurrent UTI in this population and a 2% complication rate of circumcision, 4 circumcisions would need to be performed to prevent 1 UTI.[68] On the other hand, given a risk of 1% in normal boys, 111 circumcisions would need to be performed to prevent 1 UTI. In infants, who present with a UTI and have high grade VUR we would offer circumcision if the use steroid cream to release prepucial adhesions fails to avoid breakthrough UTI.

### 5. Discontinuation of antibiotics

The optimal duration of antibiotic prophylaxis has been as controversial as the use of the antibiotic in the first place. The decision to discontinue antibiotics in boys is a more comfortable decision as compared to girls where the risk of UTI persists and there is evidence about the deleterious effects of UTI and pyelonephritis in pregnancy. The risk of new scarring diminishes considerably with increasing age but does not reduce completely.[69,70] Coulthard believes that the risk of scarring diminishes with age not because of renal maturation but simply because the most vulnerable patients would have already scarred their kidneys in that time period.[71] He showed in an experimental study in pigs that the risk of reflux nephropathy does not diminish after renal maturation.[72]

What are the arguments in favor of antibiotic discontinuation?

1. Risk of renal scarring diminishes after 4 years of age
2. Prompt treatment of PN prevents scar formation
3. VUR resolution continues beyond 5 years of age at least until adolescence

What are the prerequisites for discontinuing antibiotics?

1. Toilet trained
2. Verbally communicative
3. No evidence of LUTS, good voiding habits and no constipation
4. Parents who are capable of seeking early medical intervention if signs and symptoms of UTI develop. We give urine culture cups to the parents with an antibiotic prescription at the time of stopping antibiotics. If they suspect a UTI, a urine specimen is obtained and the parents start therapeutic antibiotics while waiting for a culture and before seeking medical advice.
5. Parental consent after being informed about the risks and benefits of antibiotic discontinuation.

Cooper *et al*., followed 51 children (mean age 8.6 y) off antibiotics and noted an 11.8% rate of intercurrent UTI with a mean follow up of 3.7 years.[73] 5 of the 6 patients with grade III VUR developed PN but none showed scarring on follow up US. Al-Sayyad *et al*. followed 78 toilet-trained children with grade I-III VUR (75% grade II) who were taken off antibiotic prophylaxis at a mean age of 5.7 years.[74] With a mean follow up of 37 months, 9 girls presented with a UTI, which was diagnosed as PN in only 1. None of the children developed any new scarring on US. Thompson studied 196 patients and included children with more severe grade IV and V VUR who were taken off antibiotics and compared to a group of children who continued prophylaxis.[75] The rate of UTI per patient per year was 0.29 on and 0.24 off antibiotics. The new onset renal scarring rate on DMSA scans was 2.6% on prophylaxis versus 3.6% off prophylaxis. Georgaki-Angelaki *et al*., discontinued antibiotics in children who had been infection free on prophylaxis for at least 2 years, had normal voiding patterns, no HN or new scarring.[76] Any acute febrile episode was treated as a PN episode in the first 24 hours until the urine cultures came back negative. With a mean follow up period of 4.4 years 8 episodes of UTI were documented in 54 children. No new scars were noted on follow up DMSA scans and the incidence of UTI was similar to the time period when these same children were on antibiotic prophylaxis. It must be mentioned that all the above studies are retrospective on older children with normal voiding habits and low to moderate VUR.

We discontinue antibiotics for all grades of VUR after the child is toilet trained and have no evidence of LUTS (assessed by history, voiding diary and uroflowmetry). Careful counseling is done to ensure that the parents maintain a low index of suspicion for a UTI and seek medical treatment soon after the onset of symptoms. A follow up US, but no VCUG is performed in a year after stopping antibiotics. In girls we advise a revisit at the post pubertal stage to review the VUR status and consider intervention.

If there is an episode of PN after cessation of antibiotics we recommend surgical intervention. If the episode of UTI is cystitis with no evidence of scarring on US/DMSA scans we consider the option of continuing off antibiotic prophylaxis again after ensuring good voiding habits.

### 6. Is VUR detrimental in adulthood and during pregnancy?

Smellie (1998) studied 226 adults with a history of VUR.[77] At initial presentation with a UTI, 68 had grade III-V VUR and 85 had renal scarring. VUR was persistent in 63. No new scars developed after puberty. The incidence of clinical PN was 17% in the group with persistent VUR in comparison with 5% in the group without VUR. In women with persistent VUR in pregnancy, there was a higher risk of febrile UTI's in women with scarred kidneys when compared to those without scarring. However, Mansfield *et al*. showed that the risk of UTI was higher in women who had undergone reimplantation compared with a similar group who had not undergone surgery.[78] Bukowski studied 77 pregnancies in 41 women who underwent antireflux surgery in childhood.[79] There was a 23% incidence of cystitis and 6% PN.

Our current indications for surgical treatment in primary VUR include

Absolute indications

1. Break through febrile culture proven UTI
2. New scars diagnosed on US or DMSA scan
3. Non-compliance with prophylaxis and/or follow up.

Relative indications

1. Febrile UTI after antibiotic discontinuation of antibiotics
2. Persistent bilateral Grade IV-V VUR
3. Persistent VUR in girls after puberty
4. Parental preference
5. Solitary kidney with persistent high grade VUR and evidence of renal scarring

## CONCLUSIONS

VUR is a heterogenous disorder and its treatment remains one of the most controversial problems in pediatric urology. There is realization that rather than a disease entity; VUR is a marker of overall urinary tract dysfunction, which may predispose to UTI. Our goal should remain the preservation of renal function and prevent the relatively small percentage of acquired renal “scarring” associated with VUR recognizing the fact that VUR is likely only one of the risk factors for development of renal scarring and UTI. The final chapter on the management of VUR is far from written and pediatric urologists have to provide a lead in conducting meaningful prospective randomized controlled studies, which define the role of diagnostic studies and interventions in children with VUR. Till that point, the current management of VUR will focus on antibiotic prophylaxis and surgical treatment for based on the classical traditional indications of surgical intervention. It is also important to resist the temptation to alter these indications in the face of an alternative minimally invasive method of surgical correction using endoscopic therapy without appropriately conducted studies demonstrating its benefit.
